# The development of high-density aggregation spatial distribution patterns under high stress

**DOI:** 10.3389/fpls.2026.1738990

**Published:** 2026-03-19

**Authors:** Kangkang Mi, Jiejun Li, Xiaoge Tian, Yuyang Song

**Affiliations:** 1 Department of Forestry, Agricultural College, Shihezi University, Shihezi, Xinjiang, China; 2 Forestry and Grassland Resources Monitoring Center of Xinjiang Production and Construction Corps, Urumqi, China; 3 Shihezi Institute of Landscape Architecture, Shihezi, Xinjiang, China

**Keywords:** ecological niche expansion, high-density aggregation, plant interaction, plant self-organization, spatial distribution patterns

## Abstract

In high-stress environments such as arid deserts, plant populations often adapt to harsh conditions by forming spatial patterns of high-density aggregation. However, most studies have focused on the static description of spatial patterns or the correlation analysis at a single time point, lacking long-term continuous observations on how high-density aggregation patterns gradually form and develop over time. Focusing on *Haloxylon ammodendron* in the Gurbantunggut Desert, we conducted five consecutive years of field surveys and spatial analyses to investigate how its aggregation pattern develops under wind erosion stress. Research has found that in the early stage of growth, the population of *H. ammodendron* forms a stable structure through high-density aggregation. As the population ages, it exhibits a pattern of gradual expansion outward from the fulcrum, with the direction of expansion being largely consistent with the main wind direction (northwest). The Random Forest model (RF) and the Generalized Linear Mixed Model (GLMM) indicate that density, wind speed, and neighbor effect are the key factors affecting population survival and spatial expansion. Wind speed modulated directional survival, shaping spatial occupancy, while neighbor effects adjusted population structure to facilitate stable expansion. Further research reveals that *H. ammodendron* actively constructs a microenvironment through self-organizing behavior, not only alleviating wind erosion stress but also achieving the expansion of ecological niches rather than the contraction as traditionally believed. This study highlights the significance of biological interactions and environmental stress jointly driving the spatial self-organization of vegetation, providing a new perspective for understanding the adaptation mechanisms of plant populations in extreme environments.

## 1 Introduction

The functioning of many resource-limited ecosystems is facilitated through spatial patterns, but the interpretation of a pattern requires a good understanding of its structure and underlying biophysical processes (Kaestner et al., 2025). The development of spatial distribution patterns under high stress typically emphasizes adaptation strategies to the environment and the interaction relationships among plants (Ben-Said et al., 2022). Due to the influence of biotic and abiotic factors, in a high-stress environment, the vegetation pattern may exhibit more fragmented or aggregated characteristics. However, plants can adapt to their environment by expanding their ecological niches, thereby mitigating the adverse effects of stress.

Growing evidence supports the idea that the formation of plant high-density aggregation arises from a complex interplay of key processes, for example, facilitative and competitive plant-plant interactions (Valiente-Banuet and Verdú, 2013). These ecological processes will occur in different non-random configuration forms and leave recognizable footprints on the spatial pattern of plants (Peralta et al., 2023). In alpine areas, it has been revealed that the intraspecific promoting effect facilitated the aggregation pattern of the *Empetrum* population and accelerated its reconstruction in the high mountain compost heap studied (Sulavik et al., 2024). Prior studies have suggested that the expansion of ecological niches can provide plants with more living space and resources, while positive interactions can enhance plants’ adaptability in adverse conditions, thereby further promoting the expansion of ecological niches (Silvertown, 2004).

Compared with the role of biotic interactions, niche expansion under environmental stress (e.g., prolonged drought, aeolian erosion) has received less attention. Studies of spatial aggregation within populations demonstrate that the strength and sign of plant interactions depend on stress. The stress−gradient hypothesis (SGH) predicts that facilitation should prevail under intense stress (Zhang and Tielbörger, 2020). In addition, other investigations have explored the correlation between the formation and development of spatial patterns and the surrounding abiotic environment (Maestre, 2006). Nonetheless, there has been a limited exploration of the concurrent impacts of both biotic, abiotic factors and others on the development of high-density agglomeration spatial patterns within a unified analysis. Moreover, many biotic interactions are sensitive to environmental factors, and this sensitivity might have unpredictable effects on altering the structure and pattern distribution of plant populations through influencing the growth and death of plants (Soliveres et al., 2012; Lynn et al., 2019). In actuality, biotic interactions might underestimate the direct influences on changes in spatial patterns on the response to environmental stress (Staniczenko et al., 2017).

In the arid desert ecosystem, variation in stress has the potential to be a major axis in spatial niche expansion and can significantly affect population dynamics. Up to now, however, little is known about how variation in environmental stress influences the development and dynamics of high-density aggregation patterns, particularly in the case of arid desert areas. Recent studies have shown that wind erosion, resulting from wind dynamics and erodible bases, appears to be more critical than other stress factors in shaping spatial patterns and plant growth during the growing season (Luo et al., 2018). Additionally, the responses of plant species to stress gradient variability vary significantly, and it seems that seed-reproducing species are more susceptible to wind erosion than clonally reproducing ones. The influence of stress, such as drought and soil salinization, on the spatial pattern of plant populations has been deeply studied, but wind erosion stress, as an important factor promoting the development of high-density aggregation patterns, has rarely been discussed (Zhang et al., 2012; Yang et al., 2024).

Species with different reproductive methods respond differently to the internal and external factors that drive the development of population spatial patterns (Chaparro-Pedraza et al., 2024). Asexually reproducing species can enable plants to rapidly expand their population size in a short period of time and occupy favorable habitats, usually covering a larger geographical area than sexually reproducing species (Kirchheimer et al., 2018). Sexually reproducing plants usually spread over long distances through seeds, which can enhance the adaptability and evolutionary potential of the population. In addition, compared with plants that reproduce asexually, plants that reproduce sexually have a wider ecological niche and can adapt to a wider range of environmental conditions. Therefore, species’ reproductive strategies must be considered when determining the main reasons for the development of plant spatial patterns. Recent research has shown that *S. alterniflora* can gather in high density through asexual reproduction and form a concentric circle distribution through the Allee effect, which is a result of spatial self-organization in the salt marsh system. This can capture more resources, increasing plant survival (Zhao et al., 2021).

Despite extensive work on plant spatial patterns, the mechanisms by which high−density patterns develop under strong wind−erosion stress remain poorly understood (Fan et al., 2018). In some systems, populations spontaneously form regular patterns to cope with stress. These studies all emphasize the role of localized ecological interactions in generating striking large-scale spatial patterns in ecosystems. The most common example is in dryland ecosystems, in the harsh environmental conditions of drylands, established vegetation improves the local environmental conditions and alters the redistribution of resources—in particular water—from bare areas to vegetation patches, which promotes the spatial aggregation of plants (Rodríguez‐Lozano et al., 2025). Current self-organization models emphasize water-mediated feedback, but neglect aeolian processes as key drivers in hyper-arid systems.

The survival rate of individuals within a population and the range of space they occupy can vary significantly depending on their age. Usually, the survival rate of newly replenished seedlings is lower compared with that of the settled individuals in the population, while the seedlings have brought about ecological niche expansion for the population, occupying more space and resources. Moreover, the characteristics of stress can determine the direction of population development. Recent research found that an adaptive strategy to wind erosion is directional growth and dispersal, allowing them to optimize resource acquisition and selectively colonize favorable microsites—as observed in *C. arborescens* under wind erosion (Luo et al., 2018). As a result, relevant age stage-spatial distribution features should be included to study the differences in the development of abiotic variables and biological variables in high-density aggregation spatial patterns. This is a vital step in understanding how the population spatial pattern responds to high environmental stress. *Haloxylon ammodendron*, a stress-tolerant small tree, is a keystone sand-fixing species widely distributed in the deserts and Gobi of northwestern China. In extreme habitats such as the Gurbantunggut Desert, its remarkable resistance to drought and salinity allows it to thrive as a dominant species. Crucially, it often persists in highly stressful microsites (e.g., dune crests) by forming high-density aggregated populations. These clusters not only enhance individual survival but also give rise to self-sustaining vegetation patches that mitigate wind erosion, stabilize sand, and locally improve soil and microclimate. Consequently, *H. ammodendron* serves as an ideal model for studying plant adaptation and vegetation-environment feedback in arid ecosystems. Up to now, however, the mechanisms driving the development of such spatial patterns remain poorly understood. Especially in high-stress environments, plants are not only passive recipients of the environment, but they can also actively change their surrounding Microenvironment through the development of their own spatial distribution, thereby “building” a more suitable space for survival. It is worth noting that in some highly stressful ecosystems, whether plants can adapt to more extreme environments by expanding their ecological niche, and in this process, which is more important, biological factors or abiotic factors?

## 2 Materials and methods

### 2.1 Study site

**Figure 1:**
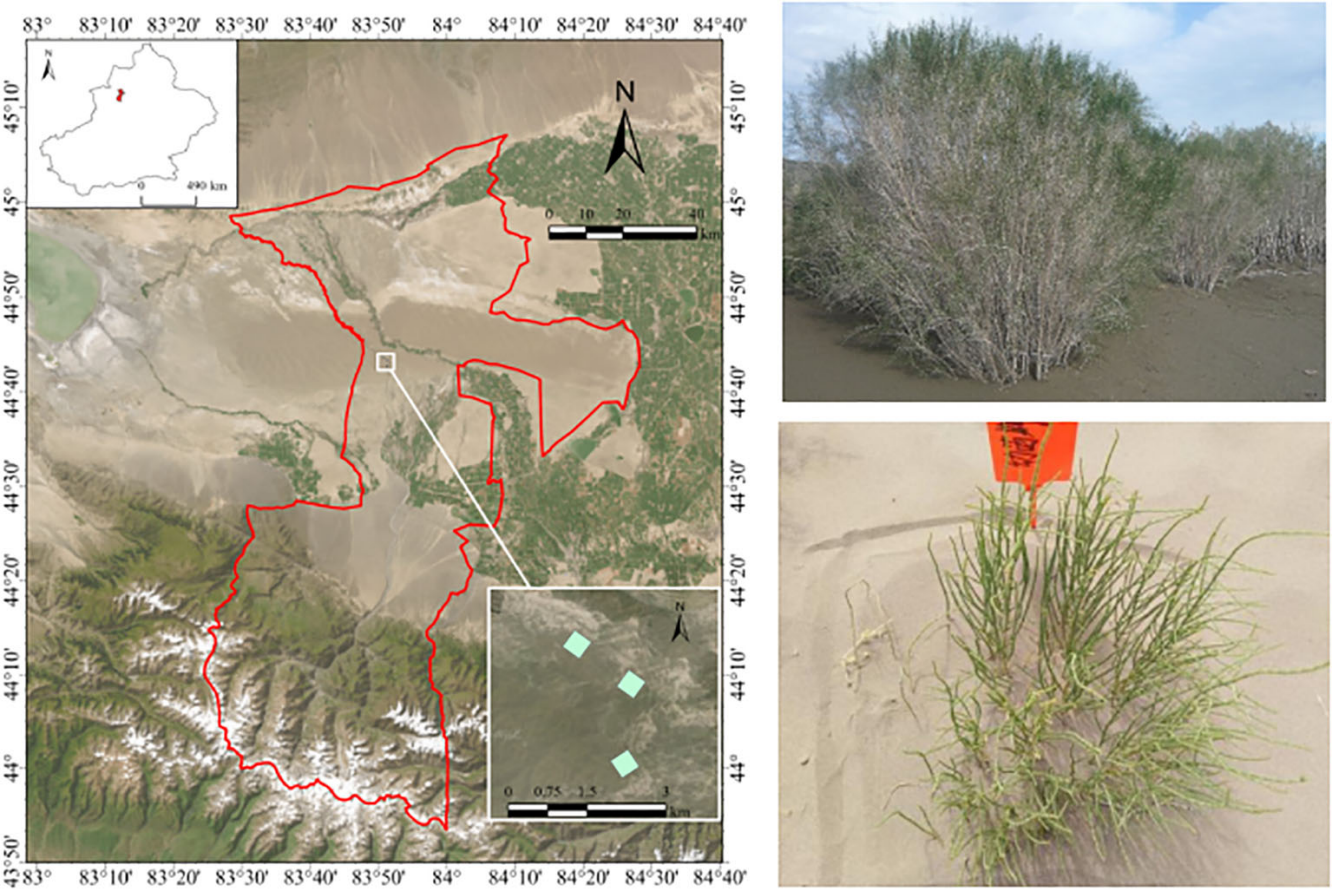
Location of the Guertu study area and field photos of *Haloxylon ammodendron* 5-year population and seedlings.

This study was conducted near Guertu, Xinjiang, China (44°15′–46°50′ N, 84°50′–91°20′ E), in the central Gurbantunggut Desert (~48,800 km²) (**Figure 1**), the second−largest desert in China. The region has a temperate arid climate with mean annual precipitation of 70–150 mm and mean annual temperature of 5.0–5.7 °C. Annual potential evaporation is 2,000–2,800 mm. Summers are hot and dry; winters are very cold. Spring mean wind speed is 9.3 m s^-1^. Ephemeral herbs dominate, with few perennial woody species (Li et al., 2023).

To examine spatial pattern development, we focused on *H. ammodendron*, a perennial woody species that tolerates drought, heat, and cold. Its deep root system allows persistence under severe wind erosion, where it often forms high−density colonies that maintain local stability. Individuals typically live 25–35 years.

### 2.2 Seedling census

To detect the growth of plants and the changes in population area, three research sites were set up in the Guertu area, Xinjiang, China, in 2018. Each plot is 400m * 400m, with a spacing of approximately 1km between plots. Within each large plot, at least 30 recently established *H. ammodendron* populations were selected as focal populations for detailed study. Each population was divided into four small plots - southeast, southwest, northeast, and northwest - based on the center point. All the plots were continuously observed for five years. The center point was selected at the center of the oldest individual in the population. The first survey was completed in November 2018.

### 2.3 Habitat variables

For each plot, we mapped all individuals, measured basal diameter, height, and calculated above-ground biomass. We tallied surviving individuals by age class and computed their mean distance to the population center. Canopy cover was estimated using vertical ground projection. Soil total nitrogen (TN) was determined by the Kjeldahl method following digestion with perchloric acid and sulfuric acid. Soil total phosphorus (TP) was measured using the molybdenum-antimony anti-spectrophotometric method after digestion with perchloric acid and sulfuric acid. Soil total potassium (TK) was analyzed by flame photometry following sodium hydroxide fusion. Soil organic carbon (OC) content was quantified by the potassium dichromate oxidation (external heating) volumetric method. Soil water content (SWC) was determined by the oven-drying method at 105 °C to constant weight. Wind speed measurements were taken during the growing season to characterize the microclimate within the population. Using a portable digital anemometer (GM816), we recorded wind speeds at multiple predetermined positions and directions across each study plot. All field measurements of the above indicators were conducted during the growth period of the *H. ammodendron* plants.

To calculate the aboveground biomass of *H. ammodendron*, this study used the biomass estimation formula of *H. ammodendron* plants in the Gurtu area by Zhao et al. (2019):


$${\text{B}}_{Guertu}=0.1238{\left(d^{2}h\right)}^{0.7492}$$

where B was the individual biomass weight, d was the basal diameter, and h was the tree height.

### 2.4 Calculation of neighboring densities

The densities of conspecific trees (Acon) directly measure the number of other individuals of the same species within a unit area or a specific radius (e.g., twice the average crown radius) around a target individual, reflecting the degree of local congestion within the population and the potential intensity of interactions among individuals. To identify the seedling and adult neighbors for each focal seedling, we calculated the densities of conspecific seedlings in every seedling plot, as well as the density of the same species of trees based on the weighted base area (BA) of the total distance of the same species of trees and within a radius of 15cm for each seedling (Kuang et al., 2017).


$$\text{Acon}=\sum_{i}^{N}BA_{i}/DIST_{i}$$

Where i is a conspecific individual (Acon), A higher Acon indicates that the spatial aggregation intensity of the population is greater. The correlation between it and the number of population survivors is a key statistical data for distinguishing the nature of the neighbor effect (net facilitation or net competition).

### 2.5 Statistical analyses

First, we collected plot−level measurements of density, the annual average wind speed during the growth period (AAWS), canopy density (CD), basal diameter (BD), height, and total branch length for individuals (TBL), and soil TK, TN, TP, organic C, PH, and moisture from 2018 to 2023 (**Table 1**). From neighbor counts and distances, we derived the neighbor index and related metrics. We then used random forests to evaluate the relative influence of 13 candidate predictors on survivor counts and occupied spatial extent. Before building the random forest model, we randomly divided all the samples into a training set and an independent test set. Among them, 70% of the samples were used to train the model, and the remaining 30% were used to verify the model’s performance. The optimal feature set was identified by recursive feature elimination with cross−validation. Variable importance was computed by permutation across training subsets and summarized across resamples (Jiang et al., 2024).

**Table 1:** Parameters of individuals of different ages and physicochemical properties of soil in a 5-year population.

		AAWS*	Acon	Height	BD	TAL	CD	Density†	TN	TP	TK	OC	PH	SWC
		(m/s)		(m)	(cm)	(cm)	(%)		(g/kg)	(g/kg)	(g/kg)	(g/kg)		(%)
Age	1	4.97±0.82	116.47±23.8	0.52±0.12	0.38±0.08	37.64±3.18	67±13	25.15±5.72	0.18±0.01	0.68±0.05	20.56±0.56	2.65±0.12	8.45±0.26	3.35±0.53
	2	5.15±0.84	106.78±18.77	0.92±0.16	0.52±0.11	55.44±4.09	73±16	19.24±4.12	0.16±0.01	0.70±0.02	20.48±0.42	2.61±0.20	8.43±0.17	3.31±0.49
	3	5.22±0.83	95±11.11	1.21±0.18	0.75±0.13	81.69±5.41	84±17	15.72±4.25	0.18±0.02	0.72±0.01	20.52±0.52	2.62±0.08	8.47±0.15	3.26±0.41
	4	5.31±0.87	66.28±6.92	1.59±0.21	0.93±0.13	89.89±5.71	91±13	13.66±3.83	0.17±0.01	0.74±0.03	20.55±0.49	2.62±0.15	8.48±0.22	3.33±0.43
	5	5.34±0.94	54.34±5.25	1.84±0.32	1.09±0.18	95.34±5.95	93±16	10.57±3.56	0.18±0.02	0.73±0.03	20.51±0.45	2.64±0.04	8.44±0.20	3.28±0.51

*AAWS represents the wind speed at the locations of individuals of different ages in the northeast direction within the population.

†Density refers to the average number of individuals of different age groups in a plant population.

We modelled survivor counts (2018–2023) and occupied extent using generalized linear mixed models (GLMMs). Count models used a negative binomial family: extent models used Gamma with a log link. Predictors were the key variables selected by random forests; we included the Wind × Neighbor and Wind × Density interactions *a priori*. Continuous predictors were Z−standardized. Plot and quadrant direction were random effects. We aggregated individuals by age class and direction when computing effect sizes, and we report the relative importance of each predictor.

All analyses were performed in R 3.6.2. We fitted GLMMs with lme4: glmer (Bates et al., 2015).

## 3 Results

### 3.1 The spatial pattern of *H. ammodendron* from 1 to 5 years old

**Figure 2:**
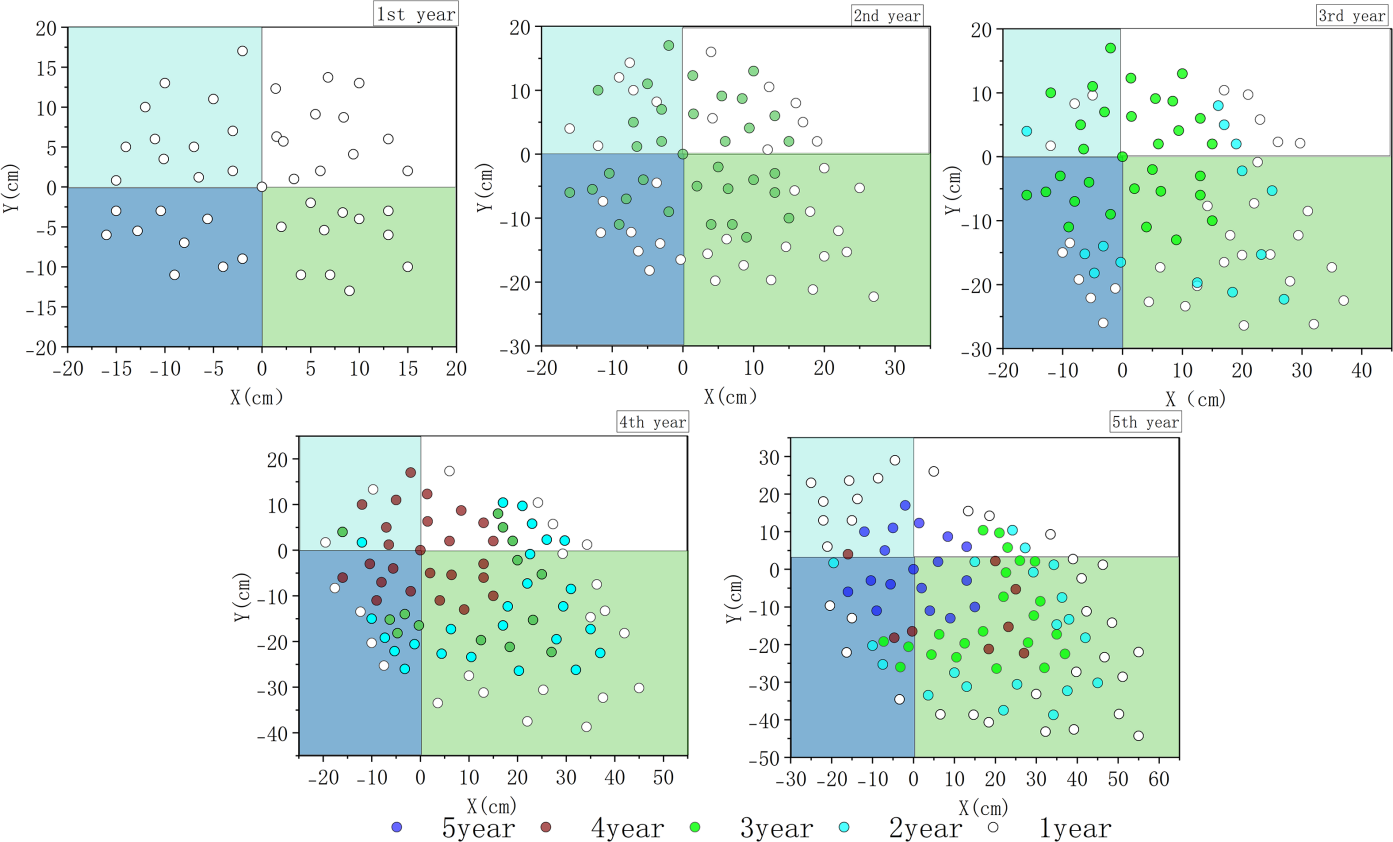
Dynamic changes in the spatial distribution pattern of *H. ammodendron* populations from 1 to 5 years. The background colors in the picture indicates different directions. Light blue represents the northwest direction, white represents the northeast direction, blue represents the southwest direction, and green represents the southeast direction.

Through continuous field investigation, we recorded the spatial pattern development of the *H. ammodendron* population over a period of 1 to 5 years (**Figure 2**). It is one of the representative samples selected. The remaining sample plots all have a similar trend to the spatial pattern development shown in **Figure 1**, as well as the survival number and spatial distribution of individuals of different ages contained in each population. The individuals of the 1-year-old population are relatively concentrated in the four directions of northeast, northwest, southeast, and southwest, with a relatively uniform distribution. The area they occupy is relatively small, approximately 35*35cm. The 2-year-old population includes both 2-year-old and 1-year-old individuals. Generally speaking, the 2-year-old individuals are in the middle position, while the newly added 1-year-old individuals are mostly located on the periphery of the 2-year-old individuals. Moreover, it has been observed that the distribution of the population in the four directions began to gradually shift. The number of surviving individuals in the northwest direction was generally larger than that in the southeast direction. The shape of the population changed from an approximate circle to an ellipse. The populations at 3, 4, and 5 years showed similar results to those at 2 years. The populations occupied new Spaces by newly replenished surviving seedlings. The number of surviving seedlings in the northwest direction was much greater than that in other directions, and the spatial distribution of the populations gradually increased along the northwest direction.

**Figure 3:**
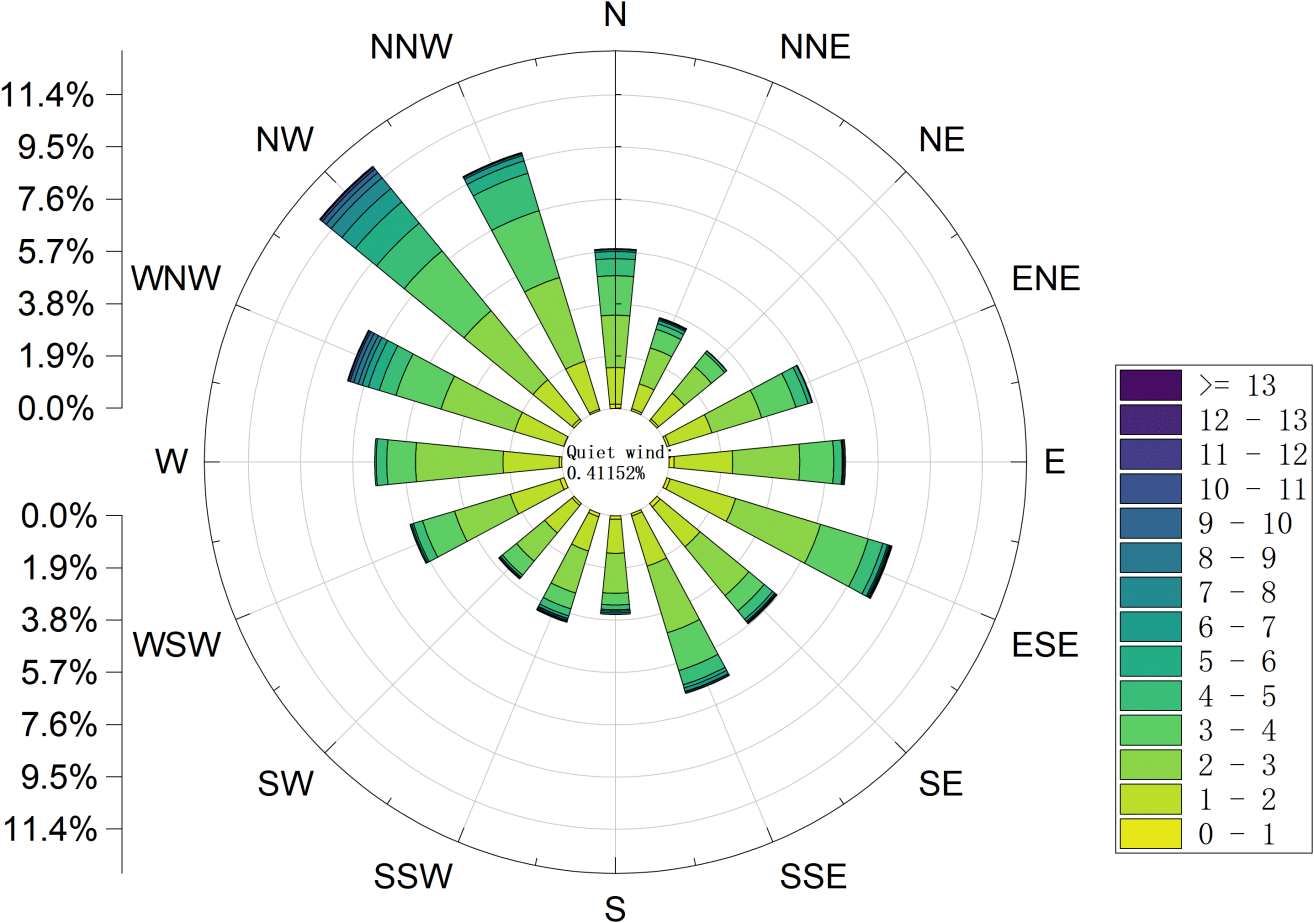
The wind speed and direction charts of the growing season (April-November) in the research area.

Among the four directions in each population, the number of surviving trees in the northwest direction is the highest, while the number of surviving trees in the southeast direction (the windward side) is the lowest. This difference becomes more significant as the population ages. However, the differences between the surviving trees in the other two directions, southwest and northeast, were not significant. In addition to the number of surviving individuals, the distance from the outermost individuals of the population to the center point of the population reflects the range of space occupied by the population. As shown in **Table 2**, the spatial range occupied by the *H. ammodendron* population in the southeast direction is significantly larger than that in other directions, and this difference also becomes more obvious as the population ages. Furthermore, it can be found that the direction of the development of the population spatial pattern is basically consistent with the direction where the wind occurs most frequently in this area, and the spatial range occupied by the newly replenished seedlings is mostly located on the leeward side (**Figure 3**).

**Table 2:** Survival quantity and spatial range in the 1-5year population across various directions.

Population age	The number of surviving trees				Distance			
	EN	SE	WS	WN	EN	SE	WS	WN
1	7.17±1.70^a^	5.48±1.27^c^	6.65±1.47^b^	6.72±1.60^b^	17.16±1.25^a^	15.96±0.78^c^	16.68±1.26^b^	16.78±1.22^b^
2	13.79±3.82^a^	11.47±3.16^c^	12.39±3.54^bc^	12.58±3.55^b^	21.20±1.40^a^	17.66±1.19^c^	19.70±1.20^b^	19.69±1.26^b^
3	18.48±4.06^a^	12.83±3.30^c^	16.15±3.76^b^	15.29±3.62^b^	38.76±2.64^a^	27.56±4.20^d^	33.94±3.75^c^	30.53±3.18^b^
4	21.34±5.74^a^	13.30±5.83^c^	18.01±5.50^b^	17.26±5.42^b^	45.6±2.53^a^	35.76±3.22^d^	40.10±2.60^c^	37.00±2.46^b^
5	27.83±6.03^a^	14.50±4.69^c^	22.17±6.23^b^	21.66±6.15^b^	66.37±3.05^a^	51.96±5.13^d^	58.77±3.64^c^	53.98±3.77^b^

Different lowercase letters in the same row indicate significant differences among different directions (P < 0.05). The values in the table are the mean ± standard deviation.

### 3.2 The random forest algorithm screens the factors that drive changes in spatial patterns

**Figure 4:**
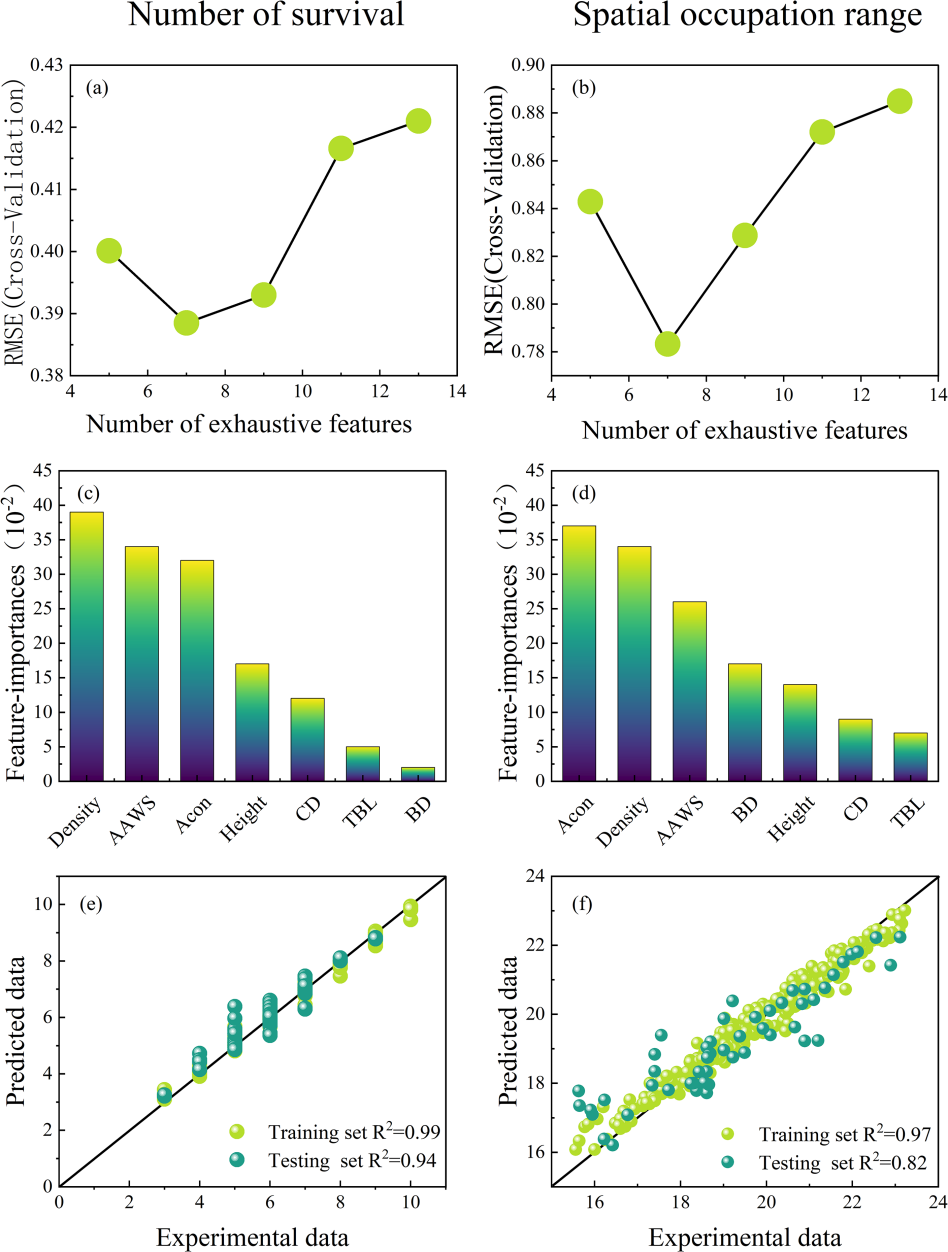
Key factors affecting survival number and occupied space range screening process and results. **(a-d)** represents cross-validation, variable screening, and variable importance ranking. The modeling effect of key factors of **(e, f)**.

The random forest model is used for exhaustive calculation; the top 7 factors determined by importance ranking and recursive feature elimination (RFE) are shown in **Figures 4a–d** (Jiang et al., 2024). Among all factor combinations for the number of surviving trees in the population, the prediction model with the combination of factors AAWS, Acon, Height, BD, TAL, CD, and Density as the input variable has the maximum coefficient of determination, R^2^ = 0.94. Among them, density, wind speed, and neighbor effect are important factors affecting the number of surviving particles, which are 39,34, and 32, respectively. Wind speed and other growth indicators are the main environmental factors influencing the spatial pattern changes of the *H. ammodendron* in the Gurbantunggut area. Soil factors were relatively less significant (Liu et al., 2022).

As shown in **Figure 4f**. For the spatial range occupied by plants, with the combination of factors AAWS, Acon, Height, BD, TAL, CD, and Density input variables, the prediction model has the maximum coefficient of determination, R^2^ = 0.82. Among them, the neighbor effect, density, and wind speed are important factors affecting the spatial range occupied by the population, which are 37,34, and 26, respectively. We refer to AAWS, Acon, Height, BD, TAL, CD, and Density as the key factors affecting the number of surviving trees in the population and occupying the spatial range. The effects of establishing a property prediction model with key factors as input are shown in **Figures 4e, f**.

### 3.3 The influence of biotic and abiotic variables on the survival quantity and spatial range occupied by plants of each age group in the population

**Figure 5:**
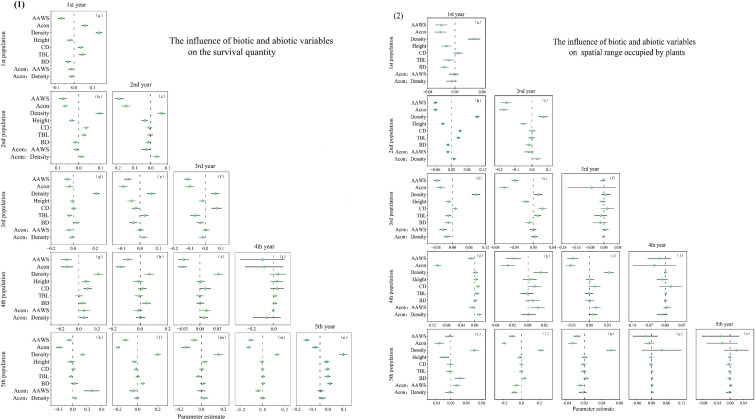
Population-wide estimates of biotic and abiotic variables on the survival quantity (1) and spatial range occupied (2) by plants of each age group.

**Figure 6:**
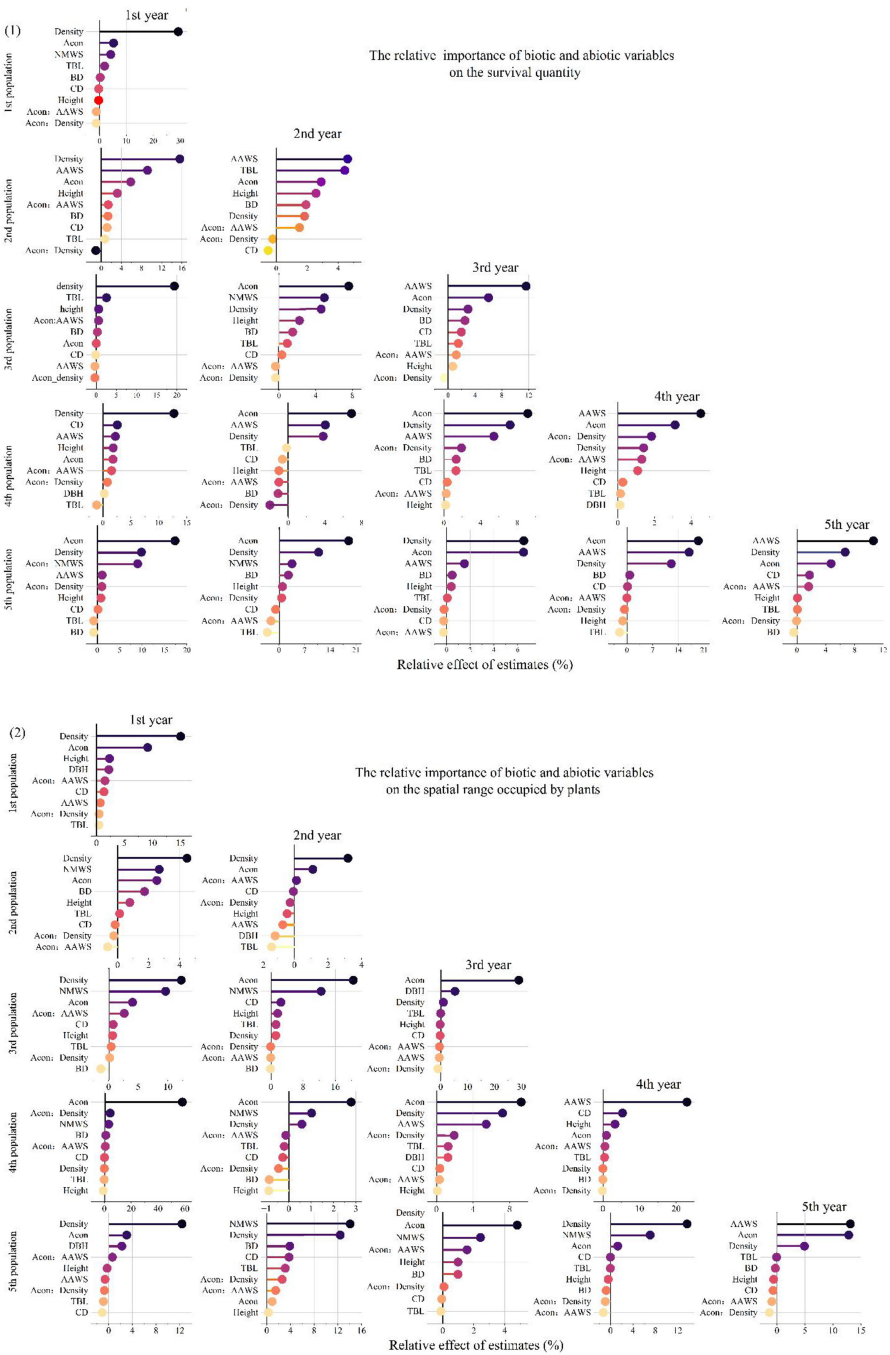
Relative importance of each factor on the survival quantity (1) and spatial range occupied (2) by plants of each age group.

By observing the changes in the spatial distribution pattern of the population from 1 to 5 years, it was found that the expansion of the population mainly relies on the number of surviving new seedlings and the spatial area they occupy. Among them, the 1-year population forms a stable spatial structure through high-density aggregation. The results indicate that, currently, density is positively correlated with both the number of surviving individuals and the range of occupied space. The influence of density on the survival of one-year-old individuals is relatively significant (29.24%), which is much more important than that of other populations (**Figure 5**). In addition, the neighbor effect also significantly influences the development of the population spatial pattern. In one-year-old populations, the neighbor effect has a significant positive effect on the number of surviving trees in the population, indicating that the greater the neighbor effect, the more surviving trees there are in the population. At this point, the positive interaction between neighbors outweighs the competition. However, this situation only occurred in a short period of time. As the population’s seedlings were replenished, differences gradually emerged among the large and small individuals within the population, and the net effect of the population changed. Competition began to take the dominant position. Furthermore, among the biological and abiotic factors screened for the newly added seedlings in the 1–5-year population, density, wind speed and neighbor effect have the most significant impact on their survival and space occupation (**Figure 6**). Specifically, wind speed, as a stress factor, has a significant negative impact on the survival of individuals of all ages in the population. Similarly, wind speed also restricts the spread of the population. Even without considering the age of individuals and the direction of the population, the analysis remains relevant.

We distinguish different age groups within the population to better explore the contributions made by individuals of each age group during the development of the population’s spatial distribution pattern. The newly replenished seedlings have occupied new space for the population, while individuals of other ages within the population are also changing in terms of the number of survivors and the range of space they occupy. Especially for those within the population’s memory, such as 2-, 3-, and 4-year-old individuals in a 5-year population, their survival and the range of space they occupy are significantly affected by the neighbor effect. Since the number of individuals of these ages does not increase but only decreases, and the neighbor effect is negatively correlated with their survival and the spatial range they occupy, it is evident that the net result of the neighbor effect currently is negative. The competition among individuals is stronger, and competition leads to the death of more disadvantaged individuals, and the occupied range also changes. Regular changes are taking place among the individuals within the population. That is, the weaker individuals within the range of different ages are beginning to die, and the population pattern is developing towards a more regular structure. Consequently, the population’s age structure shows a distinct centric zonation, decreasing radially from the core to the periphery. The number of individuals of different ages in the population also decreases as age increases. The newly added individuals can occupy new spatial areas. Those in the northwest direction are in the leeward direction, and the wind speed is effectively reduced by the blocking effect of other individuals. Therefore, the newly added seedlings in the northwest direction can obtain a better growth environment, which is the main direction of the development of the population spatial pattern.

## 4 Discussion

A changing environment significantly influences the development of plant spatial patterns through various mechanisms, and its effects on the ecological niche have been well documented throughout the world (Huang et al., 2024). Increasingly frequent extreme climate events have further highlighted the interplay between stressful environments and biotic interactions. Abiotic factors typically restrict plant distributions by defining fundamental survival conditions (i.e., the fundamental niche), while finer-scale habitat heterogeneity and biotic interactions further refine realized distributions (Wisz et al., 2012). A core tenet of this framework is that intense environmental stress acts as a powerful filter, constraining a species’ ability to utilize resources within its fundamental niche and often leading to a contraction of its realized niche. This perspective finds concrete support in empirical studies. For example, research on wild relatives of crops in Indonesia predicts that under climate change stress, the distribution ranges of multiple species (such as *Artocarpus sepicanus* and *Ficus oleifolia*) will significantly shrink, directly demonstrating how climate stress acts as an environmental filter that reduces their realized niches (Rahman et al., 2023). Such distribution shifts illustrate a broader pattern in which spatial structure dynamically reorganizes under stress. Indeed, vegetation patches may shift directionally along environmental gradients, and differential stress can produce organized patterns—such as concentric rings or banded distributions—as species sort according to tolerance (Shi et al., 2021).

Long-term environmental changes (such as climate change and geological transitions) are the main driving forces for species evolution, new species formation, and large-scale expansion of ecological niches (Meneganzin et al., 2020). Abiotic stress acts as a fundamental template upon which vegetation spatial patterns develop and change, and in arid desert regions (e.g., the Gurbantunggut Desert), intense wind erosion stress has been widely recognized as a key factor shaping the growth and spatial distribution of plants (Luo et al., 2018). Unlike the traditional view of niche contraction under high stress, the expansion of the ecological niche of *H. ammodendron* under wind erosion stress stems from its positive feedback loop of “stress-aggregation-improvement”. This self-reinforcing cycle, which varies with population age, effectively extends the species’ realized niche in a high-stress environment. Crucially, the capacity to form and sustain high-density clusters under conditions that are lethal to solitary individuals is a definitive manifestation of niche expansion. It represents a fundamental shift in the species’ ecological strategy, enabling access to and persistence within extreme habitat domains that lie outside the fundamental niche of its non-aggregated form. Our 5-year monitoring data directly supports this stress-mitigation and niche-expansion mechanism through key observations: First, the wind-shielding effect of the aggregated population is verified by microclimate data: the average wind speed within 5 year aggregated patches (5.34 ± 0.94 m·s^-1^) is 3.4% lower than that in bare sandy areas (5.53 ± 0.61 m·s^-1^), leading to a clear survival gradient—seedling survival on the sheltered leeward side is significantly higher than on the exposed windward side. Secondly, the impact of wind speed (AAWS) on the survival rate of the population decreases with age: especially in the 5-year-old population, wind speed is the most significant factor for 5-year-old individuals, accounting for 14.24%. The importance of wind speed at the initial establishment of the population is only 0.7%, indicating that as the population ages, the height of the trees, the extent of their territory will increase significantly, and the impact of wind speed on the population has also been significantly enhanced. Ultimately, the differences in wind speed among these populations are manifested as a directional expansion: seedling survival is markedly higher in the leeward (low-stress) direction, leading the population boundary to advance in an orderly, leeward-biased “frontline” pattern along the prevailing northwest wind, rather than through random diffusion. Furthermore, Yang et al. (2024) discovered in their study of coastal saline-alkali land that soil salinity gradually increased from the center of the vegetation patches, forming a distinct concentric pattern. This pattern not only directly reflects the environmental gradient (salinity) but also reflects the selection and sorting of species along the salinity gradient - the halophyte *Suaeda salsa* occupies the high-salinity edge, while more competitive species such as *Imperata cylindrica* occupy the center of the patches with lower salinity. This is similar to the aggregated pattern formed by *H. ammodendron* along the wind erosion gradient in this study, indicating that in stressful environments, environmental gradients often serve as the ‘template’ for the formation of spatial patterns, driving the development and coexistence of species’ spatial distribution (Yang et al., 2024). Overall, these findings underscore that environmental stress profoundly shapes plant spatial patterns, driving strategies that enhance fitness under adverse conditions.

Plant interactions are a major force shaping community structure and dynamics, which in turn govern population regeneration, succession, and spatial distribution patterns (Armas and Pugnaire, 2005). As highlighted by Bertness and Callaway (1994), under conditions of high environmental stress and limited resources, positive interactions (facilitation) may outweigh competition in driving community assembly. Unlike the relatively static nature of the abiotic environment, biological interactions actively modify local conditions, thereby translating fundamental niches into realized ones. Our study provides a salient case: for the *H. ammodendron* in high-stress deserts, the interaction among plants, especially facilitation, is not only a supportive interaction but also the core architect of its spatial pattern and ecological niche dimensions. The research by Alados et al. (2017) offers broader evidence that plant-plant interactions drive spatial patterning, showing that in subalpine habitats, facilitation significantly enhances the formation of aggregated vegetation patterns, whereas in semi-arid habitats, increased bare soil area tends to randomize spatial structure. This indicates that the role of plant interactions in shaping spatial patterns is habitat-specific and modulated by both long-term environmental adaptation and short-term disturbance processes (Alados et al., 2017). This role of facilitation offers a nuanced perspective on classical ecological theory. Grime’s CSR strategy theory often predicts that stress-tolerant (S) species, adapted to harsh conditions, benefit less from facilitation and are more constrained by biotic competition. However, for *H. ammodendron*, as a stress-tolerant species, it not only benefits profoundly from facilitation—evidenced by the strongly positive neighbor effect crucial for seedling survival—but also actively orchestrates it through high-density clustering. Especially on the periphery of population expansion, newly replenished seedlings are more affected by the neighbor effect(**Figure 6**). Within the established population, the balance between mutual benefit and competition helps to regulate the individual spacing and the size structure of the population, which is key to the development of the observed, orderly spatial pattern. Thus, in this system, facilitation transcends the traditional role of merely alleviating stress within a fixed fundamental niche. Instead, it serves as an active mechanism for niche construction and expansion. Biotic interactions here do not just modify a pre-defined niche; they actively push its spatial and ecological boundaries, allowing the population to occupy and stabilize terrain that lies beyond the limits of its solitary form. This highlights that in extreme environments, the capacity to generate and leverage positive feedback through spatial self-organization can be a defining trait of successful stress-tolerant strategists. Abiotic factors determine the possible locations where life may exist, but promotion determines the locations where life thrives and the size of the space it occupies. In this system, plant interactions can overcome abiotic limitations, and biological factors can regulate the expansion of population niches.

In arid desert ecosystems, quantifying the relative contributions of biotic and abiotic variables to plant survival has long been a core challenge and a subject of persistent debate among ecologists. This study starts with the intraspecific neighbor effects of plants and further extends to a broader range of biotic factors, including density, plant height, basal diameter, and canopy closure. Meanwhile, it is found that the explanatory power of biotic factors centered on neighbor effects for plant survival rate and spatial occupancy range varies across different age classes. To further clarify the regulatory role of biotic factors in this context, in high-stress ecosystems, besides neighbor effects, other biotic factors also regulate population spatial dynamics. Among these variables, population density emerges as the core driver as it governs the acquisition, partitioning, and competitive intensity of light resources. Within the population, it synergizes with other factors to collectively drive dynamic changes in population survival number and spatial occupancy range. The functional mechanisms of these factors are not isolated; instead, they generate synergistic or antagonistic effects through the light competition network. A typical example is the coupling of density and canopy closure: increased density accelerates canopy overlapping, which in turn intensifies light interception in the upper layer and reduces light availability for understory seedlings, ultimately shaping the survival patterns and spatial distribution characteristics of populations. Under high-stress conditions, *H. ammodendron* populations exhibit a high-density aggregated distribution pattern from the initial stage of establishment, with population density continuously increasing during the 1–5-year-old growth stage. The positive interactions derived from such high-density aggregation (e.g., wind erosion resistance and microhabitat improvement) not only facilitate seedling colonization but also optimize the light environment by reducing excessive solar radiation, thereby alleviating photoinhibition for young individuals. Further correlation analysis confirms a significant positive correlation between density and both population survival number and spatial occupancy range (**Figure 5**), which reveals that high-density aggregation constitutes the core basis for enhancing plant survival capacity in high-stress environments. In contrast to the dominant effects of density and neighbor interactions, the effects of other biotic factors (e.g., basal diameter and plant height) on population survival number and spatial occupancy range are less pronounced. Moreover, when compared with key abiotic variables in arid deserts, these morphological traits are of lower importance for the expansion of population spatial patterns. A distinct age stratification exists within the population, with significant differences in plant height and basal diameter among individuals of different ages: taller individuals can capture more light resources, while reducing wind speed and providing shading shelter for understory plants. Basal diameter reflects the resource competition ability of individuals and their soil consolidation effect, playing a crucial role in maintaining the stability of population spatial structure. The canopy closure of the population increases gradually with growth (**Table 1**), which records a 1.39-fold increase from the 1-year-old to 5-year-old stages, and its effects on individual survival number and spatial occupancy range are inconsistent across age classes. Specifically, canopy closure is positively correlated with the survival of 1-year-old individuals, serving as a key driver for population niche expansion. Beyond canopy structure, individual morphological traits also affect light resource acquisition, as the shoot length and branch number of Haloxylon ammodendron individuals are important guarantees for their stable acquisition of light resources: the total shoot length of the population can characterize its main growth direction, while the total shoot length in different directions reflects the spatial expansion potential of the population in the corresponding dimensions. Beyond these factors, we also examined the effects of interactions between major variables on population survival number and spatial occupancy range. It is evident that the interactions among these factors are less important than individual factors in driving the development of population spatial patterns. From an ecological mechanism perspective, this pattern of “dominance by individual factors and supplementation by interactive effects” also represents a simplified adaptive strategy for *Haloxylon ammodendron* populations to cope with arid desert environments. Plant reproductive strategies (sexual and asexual) play a key role in shaping the spatial distribution and environmental adaptability of populations (Bisang et al., 2023). Asexual reproduction enables rapid formation of dense communities through vegetative propagation, suited to stable habitats. In contrast, sexual reproduction promotes genetic diversity and stress resistance through recombination, supporting adaptation to changing conditions. Seeds dispersed by wind, water, animals, or machinery facilitate colonization from local to broad scales, directly extending population range (Leon-Osper et al., 2020). In simple terms, reproduction mode is a key strategy for plants to expand, occupy, and maintain their distribution, while the existing environment shapes and selects the most suitable reproductive strategy. For instance, animal-dispersed seeds often generate clustered distributions around nurse plants or perches, while Water-borne seeds may establish linear patterns along watercourses (Sanaa et al., 2016). Wind is the key external force driving the propagation of *H. ammodendron* seeds. Its seeds have wings and can spread over medium and long distances with the help of the wind. Seeds from the windward direction will scatter within the population. Intense competition makes it difficult for seedlings to settle down here. Some seeds from the leeward direction will scatter into the favorable environment created by the population for settlement and growth. This results in a phenomenon where the age of the population generally decreases from the center to the periphery. The small-scale aggregation of the *H. ammodendron* population is the result of limited seed diffusion caused by the separation of the winged perianth within a short period of time. (Wang et al., 2016). The reproductive strategies of Caragana arborescens at different locations on dunes provide a fascinating example: on the wind-eroded top and windward slopes of the dunes, vegetative reproduction (clonal growth) is the dominant form, which is conducive to the rapid formation of an interconnected network resistant to wind erosion; while on the windward slopes with deeper sand burial and the inter-dune areas, the proportion of sexual reproduction (seedlings) increases, to take advantage of the relatively stable conditions there for genetic renewal. This ‘specific reproductive strategy differentiation based on habitat’ indicates that plants can sense the type and intensity of micro-environmental stress and flexibly adjust their reproductive investment, thereby forming the most suitable distribution pattern in heterogeneous environments (Luo et al., 2018). This is in line with the strategy of safflower, which relies on wind-dispersed seeds to perform targeted supplementary renewal to the population refuge area (windward side), jointly revealing that reproductive behavior strategies are the core link connecting environmental heterogeneity and the spatial distribution pattern of populations.

The spatial distribution pattern of high-density aggregation of *H. ammodendron* under high stress. It is a typical self-organizing phenomenon. The spatial distribution pattern of *H. ammodendron* is a clear manifestation of environment-coupled spatial self-organization. This phenomenon arises from dynamic and long-term self-organizing processes, driven by both the interaction among individuals and their direct abiotic environment. Rather than being solely shaped by large-scale constraints, its delicate spatial structure stems from the joint regulation of biological processes and environmental factors. Self-organization is like an architect, and the expansion of ecological niches is the goal and stabilizer of self-organization evolution. Most of the spatial self-organization patterns found and studied in stressed environments, such as fairy circles or other ring-type patterns (hereafter FCs), resulting from spatial self-organization have attracted much attention in Australian and Namibian dryland ecosystems, and concentric ring patterns in Chinese salt marshes (Pringle and Tarnita, 2017; Zhao et al., 2021). These studies all emphasize the role of localized ecological interactions in generating striking large-scale spatial patterns in ecosystems. The self-organizing model is the core engine and means for plants to expand their ecological niches. It can provide ecological niches for specific species by regulating the environment and establishing biological species relationships. At the same time, it can also enhance stress resistance and stability and consolidate the expanded ecological niches. Thus, a positive feedback loop exists where self-organization consolidates the current niche, which in turn enables further niche expansion, driving a new round of self-organization at the expanding front. The population structure is thus the emergent outcome of localized positive feedback operating within specific environmental constraints. The role of environment-coupled self-organization in vegetation patterning provides important insights into niche expansion mechanisms in extreme environments. These findings offer a theoretical basis for the conservation, restoration, and sustainable management of fragile arid ecosystems.

## Data Availability

The raw data supporting the conclusions of this article will be made available by the authors, without undue reservation.
